# Copy number variations of chromosome 17p11.2 region in children with development delay and in fetuses with abnormal imaging findings

**DOI:** 10.1186/s12920-021-01065-z

**Published:** 2021-09-01

**Authors:** Yuanyuan Zhang, Xiaoliang Liu, Haiming Gao, Rong He, Guoming Chu, Yanyan Zhao

**Affiliations:** Department of Clinical Genetics, Shengjing Hospital of China Medical University, Shenyang, China

**Keywords:** 17p11.2 imbalance, Copy number variation, Development delay, MLPA, NGS

## Abstract

**Background:**

Deletion and duplication of the 3.7 Mb region in 17p11.2 result in two syndromes, Smith-Magenis syndrome and Potocki-Lupski syndrome, which are well-known development disorders. The purpose of this study was to determine the prevalence, genetic characteristics and clinical phenotypes of 17p11.2 deletion/duplication in Chinese children with development delay and in fetuses with potential congenital defects.

**Methods:**

7077 children with development delay and/or intellectual disability were screened by multiplex ligation-dependent probe amplification P245 assay. 7319 fetuses with potential congenital defects were tested using next generation sequencing technique.

**Results:**

417 of 7077 pediatric patients were determined to carry chromosome imbalance. 28 (28/7077, 0.4%) cases had imbalance at chromosome 17p11.2. Among them, 12 cases (42.9%) had heterozygous deletions and 16 cases (57.1%) had heterozygous duplications. The clinical phenotypes were variable, including neurobehavioral disorders, craniofacial/skeletal anomalies, immunologic defects, ocular problems and organ malformations. 263 of 7319 fetuses were recognized to have genomic copy number variations. Only 2 of them were found to harbor 17p11.2 imbalance. The fetus with deletion presented with ventricular septal defect and the fetus with duplication had cerebral ventricle dilation.

**Conclusion:**

Our study highlights the phenotypic variability associated with 17p11.2 variations in China. The results further expand the phenotypic spectrum of SMS/PTLS and increase awareness of these disruptive mutations among clinicians.

## Background

Chromosome 17 band p11.2 is an unstable region that is prone to nonallelic homologous recombination (NAHR). This can produce recurrent deletion or duplication within the region and contribute to copy number variation (CNV) of corresponding gene clusters [1]. Genomic disorders map to this region are two syndromes, Smith-Magenis syndrome (SMS, OMIM #182290) and Potocki-Lupski syndrome (PTLS, OMIM #610883) [2, 3], which are caused in most cases by an approximately 3.7 Mb reciprocal deletion and duplication [4]. SMS is a well known and complex neurobehavioral disorder characterized by developmental delay, intellectual disability, sleep disturbances and self-injurious behaviors [5, 6]. Patients generally have craniofacial features [6]. While, the symptoms of PTLS are considered to be infantile hypotonia, sleep apnea, congenital cardiovascular anomalies, learning disabilities, short stature, and failure to thrive [7].

The incidences of SMS and PTLS are predicted to be 1 in 25,000 [8]. However, they are likely to be underestimated, given the poor resolution of conventional G-band karyotyping analysis in detecting subtle abnormalities. G-band karyotyping has been the method of choice for cytogenetic test, accurately detecting chromosomal aberrations larger than 5 Mb [9]. Before the utilization of array comparative genomic hybridization (aCGH) and next generation sequencing (NGS) in the clinic laboratory, multiplex ligation-dependent probe amplification (MLPA) assay was widely used to detect chromosome sub-microscopic deletions and duplications of targeted regions [10, 11]. Specifically, the MLPA P245 kit is designed to screen patients presenting with developmental delay and/or intellectual disability for 31 kinds of common microdeletion or microduplication syndromes. P245 probe mix includes 3 probes in 17p11.2 region, which are RAI1 (retinoic acid induced 1), LRRC48 (DRC3, dynein regulatory complex subunit 3) and LLGL1 (LLGL scribble cell polarity complex component 1). Ratio anomalies of these probes indicate copy number variations at chromosome 17p11.2.

In this study, we presented an overview of the genetic and clinical characteristics of Chinese patients that were identified to carry 17p11.2 imbalance by MLPA and NGS. The aim of this study was to estimate the prevalence and detailed clinical characteristics of 17p11.2 deletions/duplications in children with development delay as well as in fetuses with potential congenital defects, which could contribute to provide basic information for clinical evaluation.

## Methods

### Patients and samples

From July 2013 to December 2020, 7077 pediatric patients were enrolled through the outpatients of pediatrics and clinic genetics of Shengjing Hospital of China Medical University. The range of their ages was from 30 days to 12 years. The phenotypes were collected by consulting their medical records. Most of these patients manifested intellectual disability and/or development delay. Other phenotypes also could be seen, including congenital malformation, hypotonia and feeding difficulties, speech and motor deficits, cardiovascular defects, epilepsy, hearing impairment, craniofacial and skeletal features and behavioral issues. 7129 pregnant women (7319 fetuses), who required amniocentesis for invasive prenatal diagnosis based on guideline published by Association for Clinical Cytogenetics (Prenatal Diagnosis Best Practice Guidelines 2009 v1.00), were recruited from outpatient of obstetrics from April 2018 to December 2020. Reasons for referral were the following: abnormal ultrasound scan; carrier of a chromosomal structural rearrangement; elevated risk of aneuploidy indicated by biochemical and / or ultrasound screening, and previous chromosome anomaly. The whole peripheral blood and amniotic fluid were retrieved from clinic genetics of Shengjing Hospital of China Medical University. This study was approved by Ethics Committee of Shengjing Hospital of China Medical University. Written informed consents to participate were obtained from all of the participants in this study (written informed consent to participate of individuals younger than the age of 16 were obtained from their parents or legal guardians).

### DNA extraction

Genomic DNA was extracted from the whole peripheral blood and amniotic fluid using Automatic nucleic acid extractor (Allsheng Auto-Pure 32A) with UPure Blood DNA Extraction Kit (M2002-A32) and UPure Tissue DNA Extraction Kit (M2012-02) (BioBase Technologies Co., LTD) following the manufacturer’s instructions. The concentration of DNA was detected using a spectrophotometry method (NanoDrop 1000, Thermo Scientific, USA). A concentration of 10–50 ng*/*μL DNA was prepared for MLPA and NGS assay.

### Multiplex ligation-dependent probe amplification (MLPA) assay

MLPA assay and the subsequent results analyses were performed as previously described [12]. Briefly, The SALSA MLPA KIT P245 (MRC Holland, Amsterdam, Netherlands) were used for MLPA analysis according to the manufacturer’s instructions. PCR amplification products were separated by capillary electrophoresis using ABI 3730 Genetic Analyser (Applied Biosystems, USA). Coffalyser. Net software (MLPA Holland, Amsterdam, Netherlands) was used to analyze and give an interpretation of the raw MLPA data. The detailed information of the probes included in MLPA kits was described in the manufacture’s instructions.

### Chromosome karyotype analysis

Conventional chromosome G-band karyotyping analysis of the cultures of peripheral blood and amniotic fluid blood cells was performed as previously described [12]. Leica CytoVision (Leica, USA) was used to capture images of mitotic metaphase chromosomes. The karyotyping results were identified and described upon agreement of the two examiners, with reference to the International System for Human Cytogenetic Nomenclature (ISCN 2016) [13].

### Next generation sequencing (NGS) assay

Copy number variation was detected by next generation sequencing (NGS) based CNV-seq as previously described [14]. Briefly, 50 ng of genomic DNA was fragmented to an average size of 300 bp, and end-ligated with barcoded sequence adaptors. The DNA libraries were constructed using library prep kit (Berry Genomics), and then purified by the Purification DNA Libraries for NGS kit (Berry Genomics). The DNA libraries were quantitated using the Kapa SYBR fast qPCR kit (Kapa Biosystems), with the standard of greater than 25 nmol/L. The quantitated DNA libraries were sequenced on the Illumina Nextseq CN500 platform (Illumina Inc.) to generate approximately 6 million single-end reads of 37-bp. No less than 2.5 million unique reads were aligned to the reference genome (GRCh7/hg19) using the Burrows-Wheeler mapping algorithm, and then allocated to 20-kb bins sequentially across each of 24 chromosomes. The raw data were analyzed to evaluate chromosomal copy number as previously described [15]. Cutoff copy number values used to call duplications were set at > 2.8, and those used to call deletions were set at < 1.2.

## Results

### Molecular analysis

**Fig. 1:**
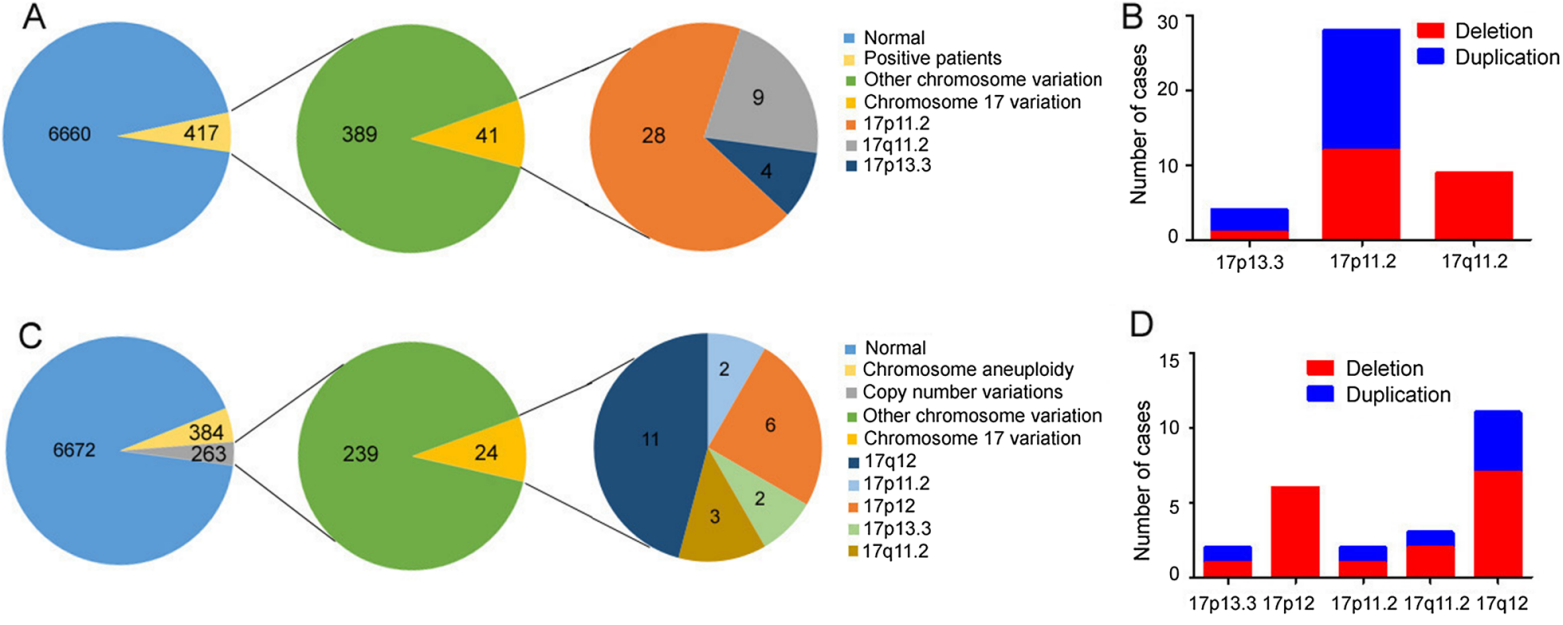
**a** The frequency of 17p11.2 imbalance in pediatric patients with development delay. **b** Numbers of pediatric patients carrying various kinds of chromosome 17 imbalances. **c** The frequency of 17p11.2 imbalance in fetuses with potential congenital defects. **d** Numbers of fetuses carrying various kinds of chromosome 17 imbalances

**Fig. 2:**
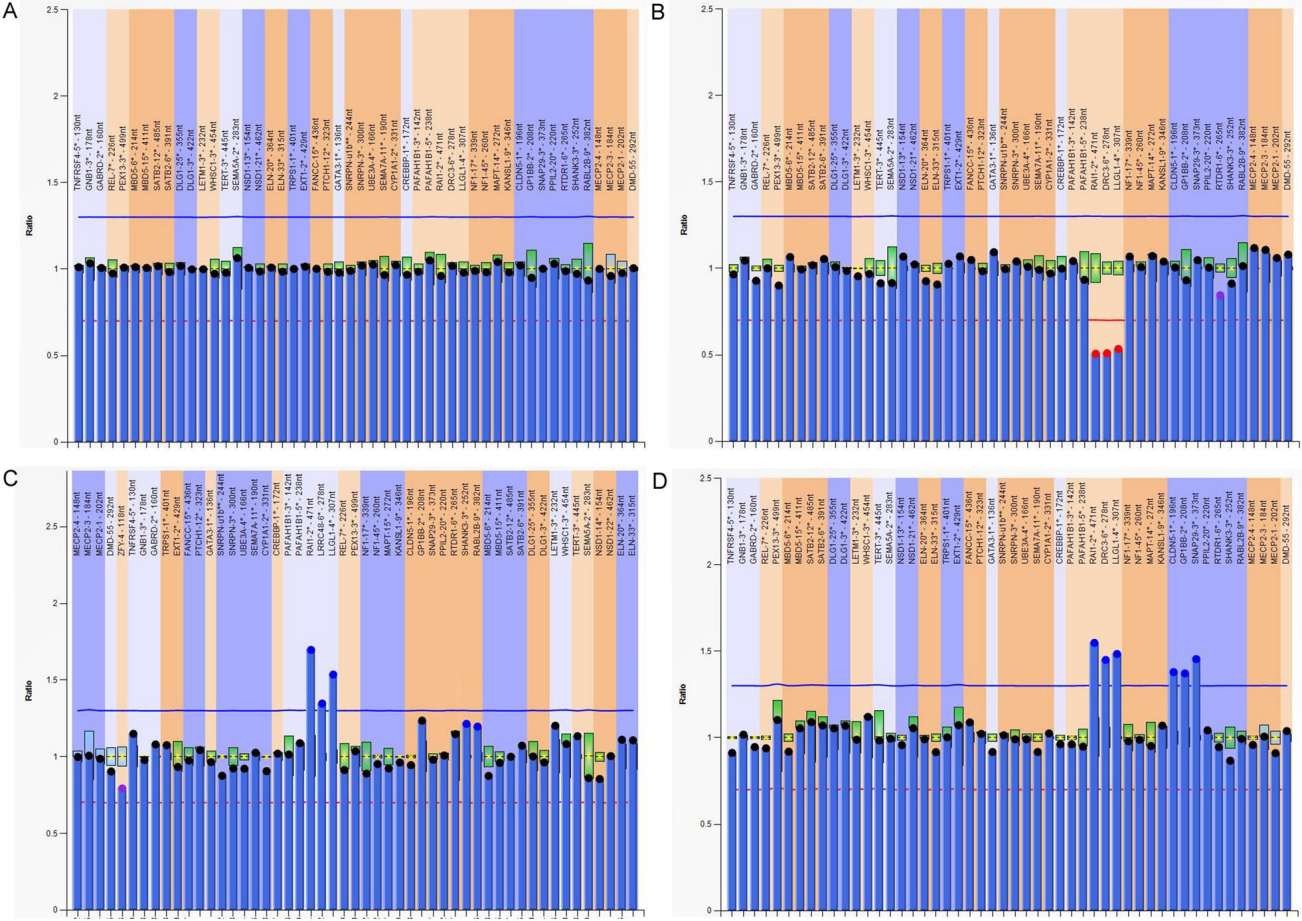
Graphs represent results of 17p11.2 imbalance analyzed by Multiplex ligation-dependent probe amplification (MLPA). X-axis represents MLPA probes. Y-axis represents probe dosage quotient. The blue line indicates probe dosage quotient of 1.35 and any probes above this line represent duplication. The red line indicates probe dosage quotient of 0.65 and any probes below this line represent deletion. The probes between 0.85 and 1.15 are considered as normal **a** A control with normal copy probes. **b** A patient carries 17p11.2 deletion. **c** A patient carries 17p11.2 duplication. **d** The patient carries duplication of both 17p11.2 and 22q11.2

**Fig. 3:**
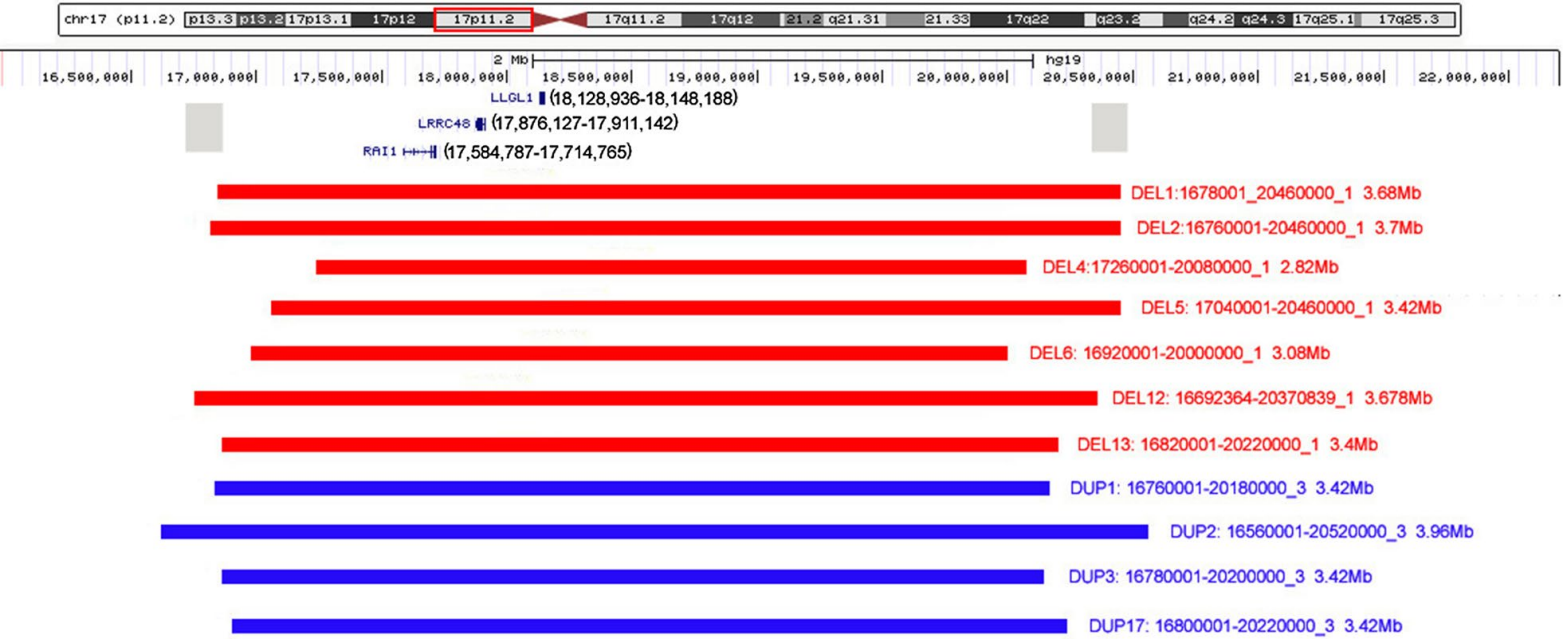
Chromosome 17p11.2 imbalance detected by NGS based CNV-seq. The region of 17p11.2 is framed by red line. Locations of RAI1, LRRC48 (DRC3) and LLGL1 genes are marked according to UCSC Genome Browser on Human Feb. 2009 (GRCh37/hg19) Assembly (https://genome.ucsc.edu/cgi-bin/hgTracks?db=hg19&lastVirtModeType=default&lastVirtModeExtraState=&virtModeType=default&virtMode=0&nonVirtPosition=&position=chr17%3A16000001%2D22200000&hgsid=1093737035_AV6lvLz0lLX1TTtV27tDLC7HUtmy). The gray squares represent the locations of low copy repeats that mediate the common recurrent deletion/duplication, which is about 3.7 Mb. Copy number deletion and duplication are represented in red and blue bar, separately. The size and breakpoint of deletion and duplication are listed on the right of the bar. DEL1 represents deletion case No.1 (sorted in Table 1); DUP1 represents duplication case No.1 (sorted in Table 2), and the like. DEL13 represents the fetus with deletion; DUP17 represents the fetus with duplication. NGS: next generation sequencing. CNV: copy number variation

MLPA P245 assay was used to screen on 7077 pediatric patients with intellectual disability and/or development delay. Totally, 417 (417/7077, 5.9%) patients were found to carry chromosome imbalance and 41 (41/417, 9.8%) patients had imbalance at chromosome 17. Among these 41 patients, 28 (28/41, 68.3%) had 17p11.2 deletions or duplications (Fig. 1a). Their ages ranged from 30 days to 10 years. 12 of the 28 (42.9%) patients had heterozygous deletions and the rest 16 (57.1%) patients had heterozygous duplications. RAI1, LRRC48 and LLGL1 probes were abnormal in all the patients with 17p11.2 imbalance. In particular, one child with 17p11.2 duplication was found to harbor three additional probes (CLDN5 (claudin 5), GP1BB (glycoprotein Ib platelet subunit beta) and SNAP29 (synaptosome associated protein 29)) imbalance, suggesting that he also had 22q11.2 duplication. By examining his parents, we confirmed that his 22q11.2 duplication was inherited from mother, while the 17p11.2 duplication was de novo. MLPA was performed on parents of the other 27 patients with CNVs in 17p11.2 and did not detect any abnormalities. The representative data of MLPA assay were shown in Fig. 2. Moreover, 9 of the 28 patients were confirmed by NGS based CNV-seq for more detailed information about sizes and breakpoints of imbalance. The results showed that the affected regions were all within chr17p11.2: 16,560,001–20,520,000 in the reference genome (GRCh7/hg19). Sizes of imbalance were mostly 3.4 Mb and 3.7 Mb, with the smallest 2.82 Mb and the largest 3.96 Mb (Fig. 3).

On the other side, 7319 fetuses with potential congenital defects were detected by NGS using DNA from amniotic fluid cells. 263 (263/7319, 3.6%) samples were found to have chromosomal aberrations, and 24 (24/263, 9.1%) of which were identified to harbor chromosome 17 imbalance. Only 2 (2/24, 8.3%) fetuses had 17p11.2 variations (Fig. 1C). One had a 3.40 Mb deletion spanning an interval of 16,820,001–20,220,000, and the other one had a 3.42 Mb duplication ranging from 16,800,001 to 20,220,000 (Fig. 3). NGS analyses of their parental blood indicated no abnormality at 17p11.2. In addition to 17p11.2, other loci at chromosome 17 were found to be affected in this fetal cohort including 17q12, 17p12, 17q11.2 and 17p13.3. Particularly, the frequencies of 17q12 and 17p12 were much higher than that of 17p11.2. And the number of cases with 17p11.2 was similar to that of cases with 17q11.2 and 17p13.3. However, among children with development delay, 17p11.2 variation was more common than 17p13.3 and 17q11.2. The numbers of cases with various kinds of chromosome 17 chromosomal aberrations were detailed in Fig. 1b and d.

### Karyotype analysis

Conventional chromosome G-band karyotyping analysis of these 30 samples with 17p11.2 deletions/duplications showed normal karyotypes as 46, XX or 46, XY. Although in some cases, genomic imbalance were suspected because the short arm of one of the two chromosome 17 was smaller than the other one, but this could also occur when the staining was not done properly or the chromosome arm was not fully extended. The results proved again that G-band karyotyping analysis was not a first-line test in the diagnosis of 17p11.2 abnormalities.

### Phenotype analysis

Among those 12 children with 17p11.2 deletions, the common phenotypes were neurobehavioral disorders, including speech and language development delay (8/12, 66.7%), gross motor development delay (8/12, 66.7%), intellectual disability (4/12, 33.3%), hyperactivity (3/12, 25%), attention deficit (3/12, 25%). Besides, individual patients also had features of autism spectrum disorder (2, 16.7%), cognitive impairment (1, 8.3%), global developmental delay (1, 8.3%), temper tantrums (1, 8.3%) and sleep disturbance (1, 8.3%). Craniofacial and skeletal features were frequently observed, with 5 (5/12, 41.7%) patients showing different appearance including wide eye distance, depressed nasal bridge, lateral canthus upwarping, square-shaped face, short stature, cleft lip/palate, tongue protrusion, antijaw, heavy eyebrows, cubitus valgus and rickets. Occasionally, some patients had immunologic defects and other manifestations, containing cardiac defects, leukodystrophy, gallstone, tracheoesophageal fistula, arachnoid cyst of greater occipital pool. Detailed clinical information was listed in Table 1.

**Table 1:** Phenotypes of 12 pediatric patients with 17p11.2 deletion

	17p11.2 deletion cases												Frequency
Case number **Gender** Age*	1 Female 7 years	2 Male 5 years	3 Male 10 years	4 Male 5 years	5 Male 5 years	6 Female 9.5 years	7 Female 30 days	8 Female 1 years	9 Male 19 months	10 Male 4 years	11 Male 3.5 years	12 Male 9.5 years	NA NA
Height (cm)^#^ ( percentile)	111 (< 3rd)	110.5 (50th)	121 (< 3rd)	113 (75th)	111 (50th)	134 (25th)	52.5 (50th)	75 (50th)	83 (75th)	103.5 (50th)	95.5 (25th)	130 (10th)	2(short stature)/12
**Neurobehavioral**													
Language delay	–	–	×	×	×	×	–	×	–	×	×	×	8/12
Motor delay	–	–	×	×	×	×	–	×	×	–	×	×	8/12
Intellectual disability	–	–	×	×	×	×	–	–	–	–	–	–	4/12
Hyperactivity	–	–	×	×	-	×	–	–	–	–	–	–	3/12
Attention deficit	–	–	×	×	-	×	–	–	–	–	–	–	3/12
Autism spectrum disorder	–	–	–	–	–	–	–	–	–	×	×	–	2/12
Cognitive impairment	–	–	–	–	–	–	–	–	–	–	×	–	1/12
Global development delay	–	–	–	–	×	–	–	–	–	–	–	–	1/12
Temper tantrums	–	–	–	×		–	–	–	–	–	–	–	1/12
Sleep disturbance	–	–	–	–	–	–	–	×	–	–	–	–	1/12
**Craniofacial/Skeletal**													
Wide eye distance	×	–	×	–	–	×	–	–	–	–	–	–	3/12
Depressed nasal bridge	×	–	–	–	–	–	–	–	–	–	–	–	1/12
Lateral canthus upwarping	–	–	–	–	–	×	–	–	–	–	–	–	1/12
Square-shaped face	–	–	–	–	–	–	–	–	–	–	–	×	1/12
Cleft lip/palate	–	–	–	–	–	–	×	–	–	–	–	–	1/12
Tongue protrusion	–	–	×	–	–	–	–	–	–	–	–	–	1/12
Antijaw	–	–	×	–	–	–	–	–	–	–	–	–	1/12
Heavy eyebrows	–	–	–	–	–	–	–	–	–	–	–	×	1/12
Cubitus valgus	–	–	×	–	–	–	–	–	–	–	–	–	1/12
Rickets	×	–	–	–	–	–	–	–	–	–	–	–	1/12
**Immunologic**													
Maxillary sinusitis	–	×	–	–	–	–	–	–	–	–	×	–	2/12
Sphenoid sinusitis	–	×	–	–	–	–	–	–	–	–	–	–	1/12
**Others**													
Cardiac defects	–	–	–	–	–	–	×	×	–	–	–	–	3/12
Leukodystrophy	–	×	–	–	–	–	–	–	–	–	–	–	1/12
Gallstone	–	×	–	–	–	–	–	–	–	–	–	–	1/12
Tracheoesophageal fistula	–	–	–	–	–	–	×	–	–	–	–	–	1/12
Arachnoid cyst of greater occipital pool	–	–	–	–	–	–	–	–	–	–	×	–	1/12

^*^ The age provided was the age at the time the sample was submitted for MLPA test. # Height was measured when the sample was submitted for MLPA test. “ × ” indicates the phenotype is present in the patient; “–” indicates the phenotype is absent in the patient; “NA” indicates not available

The 16 patients with 17p11.2 duplications presented with similar spectrum of neurobehavioral disorders to reciprocal deletions. Most patients showed gross motor development delay (8/16, 50.0%) and intellectual disability (8/16, 50.0%). The symptoms of speech and language development delay (7/16, 43.8%), global developmental delay (5/16, 31.2%) and attention deficit (4/16, 25%) were less common. Muscular hypertonia, stiff expression, hyperactivity, features of autism spectrum disorder and sleep disturbance could also be observed. Brain abnormalities and ocular defects were found in a smaller portion of patients. Two patients had craniofacial and skeletal anomalies, including one case of short stature and one case of ptosis. Two of 10 male patients were diagnosed with cryptorchidism (see Table 2).

**Table 2:** Phenotypes of 16 pediatric patients with 17p11.2 duplication

	7p11.2 duplication cases																Frequency
Case number	1	2	3	4	5	6	7	8	9	10	11	12	13	14	15	16*	
Gender	Female	Male	Male	Male	Male	Male	Female	Female	Female	Female	Male	Male	Female	Male	Male	Male	NA
Age^#^	7 years	5 months	7 years	16 months	2 years	2 years	9.5 years	9 months	2.5 years	1 years	7 years	8 months	9 years	40 days	18 months	9 months	NA
Height (cm)^$^ ( percentile)	122.5 (50th)	68 (75th)	119 (25th)	79.5 (50th)	90 (75th)	88 (50th)	135 (50th)	71.5 (50th)	90 (25th)	77 (75th)	122 (50th)	69.5 (50th)	117.5 (< 3rd)	54.5 (50th)	78.5 (25th)	70 (25th)	1 (short stature)/16
**Neurobehavioral**																	
Motor delay	×	–	–	×	–	×		×	–	×	×	–	–	–	×	×	8/16
Intellectual disability	×	–	×	×	×	–	×	–	–	–	×	–	×	–	–	×	8/16
Language delay	×	–	–	×	–	×	×	–	×		×	–	×	–	–	–	7/16
Global developmental delay	–	×	–	–	×	–	–	–	–	×	–	×	×	–	–	–	5/16
Attention deficit	×		×	–	–	–	–	–	–	–	×	–	×	–	–	–	4/16
Muscular hypotonia	–	–	–	–	–	–	–	×	–	–	×	–	–	–	–	×	3/16
Stiff expression	×	–	–	×	–	–	–	–	–	–	×	–	–	–	–	–	3/16
Hyperactivity	×	–	–	–	–	–	–	–	–	–	×	–	–	–	–	–	2/16
Autism spectrum disorder	–	–	–	–	–	×	–	–	–	–	–	–	–	–	–	–	1/16
Sleep disturbance	–	×	–	–	–	–	–	–	–	–		–	–	–	–	–	1/16
**Ocular**																	
Strabismus	–	–	–	–	–	–	×	–	–	–	×	–	–	–	–	–	2/16
Amblyopia	–	–	–	–	–	–	×	–	–	–		–	–	–	–	–	1/16
Glaucoma	–	–	–	–	–	–	–	–	–	–	×	–	–	–	–	–	1/16
**Craniofacial/Skeletal**																	
Ptosis	–	–	–	–	–	–	–	–	–	–	–	–	–	–	–	×	1/16
Others																	
Cryptorchidism	–	–	–	–	–	–	–	–	–	–	–	–	–	×	×	–	2/16
Descending cerebellar tonsil	×	–	–	–	–	–	–	–	–	–	–	–	–	–	–	–	1/16
Hydrocephalus	×	–	–	–	–	–	–	–	–	–	–	–	–	–	–	–	1/16
Arachnoid cyst of greater occipital pool	–	–	–	–	–	–	–	–	–	–	–	–	–	–	–	×	1/16

* This child had both 17p11.2 duplication and 22q11.2 duplication. # The age provided was the age at the time the sample was submitted for MLPA test. $ Height was measured when the sample was submitted for MLPA test. “×” indicates the phenotype is present in the patient; “–” indicates the phenotype is absent in the patient; “NA” indicates not available

Fetal anomalies were usually difficult to determine and mostly suggested by imaging examination. Both of the two fetuses had abnormal imaging findings when they went though antenatal care. As a result, they were speculated to have congenital defects. Specifically, the fetus with 17p11.2 deletion showed ventricular septal defect by three dimensional color Doppler ultrasound, while the fetus with reciprocal duplication had mild left ventricular dilatation detected by ultrasound as well as head CT scan.

## Discussion

Deletion/duplication of chromosome 17p11.2 can lead to SMS/PTLS, which are multiple congenital anomalies usually recognized in infancy or early childhood because of craniofacial deformity and developmental delay. In our study, MLPA P245 assay was applied to screen in 7077 cases with intellectual disability and/or developmental delay. The results revealed that 17p11.2 was the fourth frequent pathogenic copy number variations (CNVs) with a frequency of 0.40% (28/7077), following 15q11.2 (1.21%), 7q11.23 (1.19%) and 22q11.2 (0.85%). The prevalence of 17p11.2 deletions and duplications were 0.17% (12/7077) and 0.23% (16/7077), respectively, which were higher than the frequencies reported in literature (0.10% and 0.06%) [16]. Some analyses had suggested that NAHR-generated deletions occurred approximately twice as frequently as duplications [17]. While in the present study, the number of patients with duplications was more than that of patients with deletions.

Most 17p11.2 deletions and reciprocal duplications have common breakpoints, although deletions and duplications of different sizes have been identified. Both appear to involve a 2.1–4.9 Mb chromosome interval in 17p11.2 region that includes *RAI1* gene [4]. Our MLPA results showed that all the patients with 17p11.2 imbalance had RAI1, LRRC48 and LLGL1 probes abnormal. These probes located in the region of chr17: 17,681,458 to 18,244,875. NGS analyses of 9 children and 2 fetuses displayed that the affected regions were all in the range of chr17: 16,560,001–20,520,000, with the sizes of imbalance mostly 3.4 Mb and 3.7 Mb. The affected regions were largely overlapped and it was difficult to analyze the correlation between genotype and phenotype. However, the NGS data and phenotypes of the 3 patients with duplications indicated that larger duplication may be more prone to have globe developmental delay and sleep disturbance. R*AI1* gene is a main dosage-sensitive gene in this genomic interval. The protein of RAI1 is highly expressed in neurons during the early stage of neurodevelopment [18]. Haploinsufficiency or triplosensitivity of *RAI1* gene is believed to be responsible for most features of SMS and PTLS, such as behavioral, craniofacial, and neurological signs and symptoms [19, 20]. Although RAI1 was shown to be responsible for most SMS/PLTS features, other genes in the 17p11.2 region also contribute to the variability and severity of the phenotype in 17p11.2 deletion/duplication cases [21–24].

The most common phenotype in pediatric patients with deletions was neurobehavioral disorder. The frequency was 75% (9/12), which was conform to the published data (> 75%) [25]. The incidence of craniofacial / skeletal abnormalities was 41.7% (5/12), which was lower than the published data (> 75%) [25]. Two patients were found to have significant short stature (< 3rd centile); with the frequency was 16.7% (2/12), although the frequency in the published data was 50–75% [25]. In literatures, immune function abnormalities and heart defects were less common phenotypes that could be seen in 25–50% individuals [25]. While, in our study, the frequencies were both 16.7% (2/12). Tracheobronchial problem was reported to be seen in 50 -75% patients [25], but we found only one patient had tracheoesophageal fistula. Leukodystrophy, gallstone and arachnoid cyst of greater occipital pool were not reported in published data, but could be seen in this cohort. From the above comparison, we saw that the phenotypes of Chinese patients were somewhat different from those published before. Nevertheless, the phenotypic frequency of patients with duplications was rarely reported. In our study, 15 out of 16 patients had neurobehavioral disorder; with the frequency was 93.7%. It is worth mentioning that although cardiovascular abnormalities were reported in about 40% of PTLS patients [26], none were found in our duplication cohort.

It is considered that genomic duplications are generally better tolerated than deletions. Nevertheless, in our study, patients with duplications were clinically recognized earlier than those with deletions, with the average age were 3.4 vs. 5.4 except neonates. It was reported that PTLS patients can manifest with severe hypotonia and failure to thrive during infancy, and with significant behavioral abnormalities [27]. In both groups of patients with deletions and duplications, the most common neurobehavioral syndromes were motor delay, intellectual disability and language delay. Besides, patients with deletions were more prone to hyperactive and attention deficit, while global developmental delay, muscular hypotonia and stiff expression were usually seen in patients with duplications. Moreover, craniofacial features and cardiac defects were more common in patients with deletions compared to that with duplications. There were 2 of 10 male patients with duplications had cryptorchidism. While in decipher database, seven patients with 17p11.2 deletions were reported to have external genital malformation, including 4 males and 3 females (https://www.deciphergenomics.org/search/patients/results?q=17p11.2).

As far as we know, the direct and first-hand descriptions on neonatal phenotypes of SMS/ PTLS were very rare. The abnormal ultrasound findings of the fetuses with a deletion/duplication of 17p11.2 were even rarely reported. Most of the reported cases were retrospective reports of patients aged > 2 who were later diagnosed. In the present study, 2 neonates and 2 fetuses were identified. Among of them, 2 cases were referred to the doctors for cardiac defects and were detected to harbor 17p11.2 deletion. The other 2 cases with duplications had milder manifestations, that one had bilateral cryptorchidism and one had cerebral ventricle dilation. The identification of neonatal and fetal cases may increase awareness of these rare disorders among clinicians so that patients would be diagnosed earlier. An early diagnosis will lead to more timely developmental intervention and medical management, which will improve clinical outcomes.

In the cohort of 7319 fetuses with potential congenital defects, 263 cases (263/7319, 3.6%) were identified to have genomic disorders, and this number is less than that of chromosome aneuploidy (384/7319, 5.2%). 24 of 263 (9.1%) cases with chromosomal aberrations had imbalance at chromosome 17, proving again that chromosome 17 was prone to variation. Interestingly, of the distinct genomic disorders mapped to chromosome 17, 17q12 was the most frequently affected region in this fetal cohort, followed by 17p12. Few fetuses carried 17p13.3, 17p11.2 and 17q11.2 variations. However, 17p11.2 imbalance was far more common than 17p13.3 and 17q11.2 among pediatric patients with development delay. We could not get the data of 17p12 and 17q12 loci in pediatric patients, as probes of these two regions were not included in MLPA P245 kit. The reasons why 17p11.2 changes had a higher frequency in pediatric patients than fetus might be as follows. Firstly, the inclusion criteria of these two cohorts were different. Pediatric patients were those with development delay, and the fetuses were suspected to have potential congenital defects. Secondly, disorders associated with deletion in 17p13.3 are brain malformations with severe mental handicap, epilepsy and a reduced lifespan, while that of duplication in this region are mainly intellectual disability [28]. Genomic imbalance of 17q11.2 usually encompasses *NF-1* (type 1 neurofibromatosis) gene. NF-1 deletion is characterized with cafe-au-lait spots, neurofibromas and Lisch nodules in the iris [29]. Children with 17p11.2 variations had more obvious neurobehavioral features so they were suspected to have development disorders by clinicians. Finally, fetal symptoms of 17p11.2 imbalance may not be sufficiently severe to warrant a detailed examination, and hence, the prevalence may be underestimated.

## Conclusion

Chromosome 17p11.2 deletion/duplication is a relatively common genomic disorder in patients with development delay in China. We conducted a study to provide prevalence, genotype and phenotype characteristics of 17p11.2 variations in pediatric patients with development delay and/or intellectual disability and in fetuses with potential congenital defects. We hope our findings could further expand the phenotypic spectrum of SMS/PTLS and increase awareness of these disruptive mutations among clinicians. Once the diagnosis is confirmed, the patient should be referred to clinical geneticists and other allied health-care providers to get multidisciplinary management. In addition, the recurrence risks for the parents and the patients should be addressed, and the importance of family support should be highlighted.

## Abbreviations

aCGH: Array comparative genomic hybridization
CLDN5: Claudin 5
CLTCL1: Clathrin heavy chain like 1
CNV: Copy number variation
DRC3: Dynein regulatory complex subunit 3
GP1BB: Glycoprotein Ib platelet subunit beta
LLGL1: LLGL scribble cell polarity complex component 1
MLPA: Multiplex ligation-dependent probe amplification
NAHR: Nonallelic homologous recombination
NGS: Next generation sequencing
PTLS: Potocki-Lupski syndrome
RAI1: Retinoic acid-induced 1
SMS: Smith-Magenis syndrome
SNAP29: Synaptosome associated protein 29

## Declarations

### Availability of data and materials

The datasets generated and/or analyzed during the current study are available in the figshare repository (https://figshare.com/articles/figure/Data_of_17q11_2_variation/14585979).

## References

[1] Morrow EM (2010). Genomic copy number variation in disorders of cognitive development. J Am Acad Child Adolesc Psychiatry.

[2] Shaw CJ, Bi W, Lupski JR (2002). Genetic proof of unequal meiotic crossovers in reciprocal deletion and duplication of 17p11.2. Am J Hum Genet.

[3] Bi W, Park SS, Shaw CJ, Withers MA, Patel PI, Lupski JR (2003). Reciprocal crossovers and a positional preference for strand exchange in recombination events resulting in deletion or duplication of chromosome 17p11.2. Am J Hum Genet.

[4] Liu P, Lacaria M, Zhang F, Withers M, Hastings PJ, Lupski JR (2011). Frequency of nonallelic homologous recombination is correlated with length of homology: evidence that ectopic synapsis precedes ectopic crossing-over. Am J Hum Genet.

[5] Gropman AL, Duncan WC, Smith AC (2006). Neurologic and developmental features of the Smith-Magenis syndrome (del 17p11.2). Pediatr Neurol.

[6] Elsea SH, Girirajan S (2008). Smith-Magenis syndrome. Eur J Hum Genet.

[7] Potocki L, Bi W, Treadwell-Deering D, Carvalho CM, Eifert A, Friedman EM, Glaze D, Krull K, Lee JA, Lewis RA, Mendoza-Londono R, Robbins-Furman P, Shaw C, Shi X, Weissenberger G, Withers M, Yatsenko SA, Zackai EH, Stankiewicz P, Lupski JR (2007). Characterization of Potocki-Lupski syndrome (dup(17)(p11.2p11.2)) and delineation of a dosage-sensitive critical interval that can convey an autism phenotype. Am J Hum Genet.

[8] Greenberg F, Guzzetta V, Montes de Oca-Luna R, Magenis RE, Smith AC, Richter SF, Kondo I, Dobyns WB, Patel PI, Lupski JR (1991). Molecular analysis of the Smith-Magenis syndrome: a possible contiguous-gene syndrome associated with del(17)(p11.2). Am J Hum Genet.

[9] Kan AS, Lau ET, Tang WF, Chan SS, Ding SC, Chan KY, Lee CP, Hui PW, Chung BH, Leung KY, Ma T, Leung WC, Tang MH (2014). Whole-genome array CGH evaluation for replacing prenatal karyotyping in Hong Kong. PLoS ONE.

[10] Ahn JW, Ogilvie CM, Welch A, Thomas H, Madula R, Hills A, Donaghue C, Mann K (2007). Detection of subtelomere imbalance using MLPA: validation, development of an analysis protocol, and application in a diagnostic centre. BMC Med Genet.

[11] Kjaergaard S, Sundberg K, Jørgensen FS, Rohde MD, Lind AM, Gerdes T, Tabor A, Kirchhoff M (2010). Diagnostic yield by supplementing prenatal metaphase karyotyping with MLPA for microdeletion syndromes and subtelomere imbalances. Prenat Diagn.

[12] Zhang Y, Liu X, Gao H, He R, Zhao Y (2021). Identifying of 22q11.2 variations in Chinese patients with development delay. BMC Med Genomics.

[13] McGowan-Jordan J, Simons A, Schmid M (2016). ISCN: an international system for human cytogenomic nomenclature (2016).

[14] Cui W, Liu X, Zhang Y, Wang Y, Chu G, He R, Zhao Y (2019). Evaluation of non-invasive prenatal testing to detect chromosomal aberrations in a Chinese cohort. J Cell Mol Med.

[15] Liang D, Peng Y, Lv W, Deng L, Zhang Y, Li H, Yang P, Zhang J, Song Z, Xu G, Cram DS, Wu L (2014). Copy number variation sequencing for comprehensive diagnosis of chromosome disease syndromes. J Mol Diagn.

[16] Cooper GM, Coe BP, Girirajan S, Rosenfeld JA, Vu TH, Baker C (2011). A copy number variation morbidity map of developmental delay. Nat Genet.

[17] Turner DJ, Miretti M, Rajan D, Fiegler H, Carter NP, Blayney ML, Beck S, Hurles ME (2008). Germline rates of de novo meiotic deletions and duplications causing several genomic disorders. Nat Genet.

[18] Fragoso YD, Stoney PN, Shearer KD, Sementilli A, Nanescu SE, Sementilli P, McCaffery P (2015). Expression in the human brain of retinoic acid induced 1, a protein associated with neurobehavioural disorders. Brain Struct Funct.

[19] Girirajan S, Elsas LJ, Devriendt K, Elsea SH (2005). RAI1 variations in Smith-Magenis syndrome patients without 17p11.2 deletions. J Med Genet.

[20] Walz K, Caratini-Rivera S, Bi W, Fonseca P, Mansouri DL, Lynch J, Vogel H, Noebels JL, Bradley A, Lupski JR (2003). Modeling del(17)(p11.2p11.2) and dup(17)(p11.2p11.2) contiguous gene syndromes by chromosome engineering in mice: phenotypic consequences of gene dosage imbalance. Mol Cell Biol.

[21] Girirajan S, Vlangos CN, Szomju BB, Edelman E, Trevors CD, Dupuis L, Nezarati M, Bunyan DJ, Elsea SH (2006). Genotype-phenotype correlation in Smith-Magenis syndrome: evidence that multiple genes in 17p11.2 contribute to the clinical spectrum. Genet Med.

[22] Liburd N, Ghosh M, Riazuddin S, Naz S, Khan S, Ahmed Z, Riazuddin S, Liang Y, Menon PS, Smith T, Smith AC, Chen KS, Lupski JR, Wilcox ER, Potocki L, Friedman TB (2001). Novel mutations of MYO15A associated with profound deafness in consanguineous families and moderately severe hearing loss in a patient with Smith-Magenis syndrome. Hum Genet.

[23] Castigli E, Wilson SA, Garibyan L, Rachid R, Bonilla F, Schneider L, Geha RS (2005). TACI is mutant in common variable immunodeficiency and IgA deficiency. Nat Genet.

[24] Dardour L, Verleyen P, Lesage K, Holvoet M, Devriendt K (2016). Bilateral renal tumors in an adult man with Smith-Magenis syndrome: The role of the FLCN gene. Eur J Med Genet.

[25] Smith ACM, Boyd KE, Brennan C, Charles J, Elsea SH, Finucane BM, Foster R, Gropman A, Girirajan S, Haas-Givler B. Smith-Magenis Syndrome. 2001 [updated 2019 Sep 5]. In: Adam MP, Ardinger HH, Pagon RA, Wallace SE, Bean LJH, Mirzaa G, Amemiya A, editors. GeneReviews® [Internet]. Seattle (WA): University of Washington, Seattle; 1993–2021. 20301487

[26] Jefferies JL, Pignatelli RH, Martinez HR, Robbins-Furman PJ, Liu P, Gu W, Lupski JR, Potocki L (2012). Cardiovascular findings in duplication 17p11.2 syndrome. Genet Med.

[27] Soler-Alfonso C, Motil KJ, Turk CL, Robbins-Furman P, Friedman EM, Zhang F, Lupski JR, Fraley JK, Potocki L (2011). Potocki-Lupski syndrome: a microduplication syndrome associated with oropharyngeal dysphagia and failure to thrive. J Pediatr.

[28] Blazejewski SM, Bennison SA, Smith TH, Toyo-Oka K (2018). Neurodevelopmental genetic diseases associated with microdeletions and microduplications of chromosome 17p13.3. Front Genet.

[29] Corsello G, Antona V, Serra G, Zara F, Giambrone C, Lagalla L, Piccione M, Piro E (2018). Clinical and molecular characterization of 112 single-center patients with Neurofibromatosis type 1. Ital J Pediatr.

